# Size, Accumulation and Performance for Research Grants: Examining the Role of Size for Centres of Excellence

**DOI:** 10.1371/journal.pone.0147726

**Published:** 2016-02-10

**Authors:** Carter Bloch, Jesper W. Schneider, Thomas Sinkjær

**Affiliations:** 1 Danish Centre for Studies in Research and Research Policy, Department of Political Science, Aarhus University, Bartholins Alle’ 7, 8000 Aarhus C., Denmark; 2 Danish National Research Foundation, Holbergsgade 14, 1, 1057 Copenhagen K., Denmark; 3 Department of Health Science and Technology, Aalborg University, Fredrik Bajers Vej 7 D2, 9220 Aalborg East, Denmark; Universidad de Las Palmas de Gran Canaria, SPAIN

## Abstract

The present paper examines the relation between size, accumulation and performance for research grants, where we examine the relation between grant size for Centres of Excellence (CoE) funded by the Danish National Research Foundation (DNRF) and various ex post research performance measures, including impact and shares of highly cited articles. We examine both the relation between size and performance and also how performance for CoEs evolves over the course of grant periods. In terms of dynamics, it appears that performance over the grant period (i.e. 10 years) is falling for the largest CoEs, while it is increasing for those among the smallest half. Overall, multivariate econometric analysis finds evidence that performance is increasing in grant size and over time. In both cases, the relation appears to be non-linear, suggesting that there is a point at which performance peaks. The CoEs have also been very successful in securing additional funding, which can be viewed as a ‘cumulative effect’ of center grants. In terms of new personnel, the far majority of additional funding is spent on early career researchers, hence, this accumulation would appear to have a ‘generational’ dimension, allowing for scientific expertise to be passed on to an increasing number of younger researchers.

## Introduction

We know very little about the relationship between size and the duration of funding and subsequent research performance. Size is a monetary concept, for example, the size of research grants, while funds are exchanged for researchers, technical and administrative personnel, infrastructure, networking etc. Performance is the (measurable) outcome of research and very often direct funding and performance are linked together, where quality and efficiency in performance are seen as success criteria. Questions whether impact is positively related to funding size, e.g. whether larger funds produce greater discoveries are often raised, but we know very little about the role of grant size and the composition of resources behind such grants and to what extent this may influence performance [1].

Based on the results from a selective Canadian case study, Fortin and Currie [1] argue that funding strategies that target diversity, rather than "excellence", are likely to prove to be more productive. Hicks and Katz [2] argue for a somewhat contrary position. They argue that the inequality in research performance should be even more explicit when it comes to funding but no decision maker with the power to establish a distribution of public money would dare to match the level of this inequality in research performance. Hicks and Katz [2] argue that decision makers who actually increase the concentration of resources probably implement less inequality than would be justified by differences in research performance. To them, more equal distribution of funding is likely to suppress incentives for the very best scientists and the consequences for the performance of a national research system may be substantial. On the other hand, there may be diminishing returns to research due to scale effects or the risk that generous budgets are used less efficiently than tight budgets (e.g., [3–4]). For example Walsh and Lee [5] find that large centers and research teams typically have more complex bureaucratic structuring in terms of division of labor, standardization and organization. Even without changes in productivity, some studies suggest that prestigious scientists get further recognition more easily than unknown scientists [6]. To a large extent this is a well-established theoretical and empirical fact of the science system (e.g., [6–10]). The supposedly “best” researchers and units benefit from self-enforcing processes, confirming and strengthening their status, yet there are also limits to “cumulative advantages” [6,11], and various social mechanisms seemingly to some degree act to restrain unequal distributions of funding [2,12]. Obviously, this is interesting from a science policy perspective. Agencies that fund scientific research often struggle with the question: is it more effective to give large grants to a few “elite” researchers, or smaller grants to many researchers? This question and the distinction between large and small grants are further complicated however by potential generational effects of grants. Large grants will often include a number of early career researchers that both contribute to and learn from the grant research.

The purpose of the present analysis is to examine the relation between size, accumulation and performance for research grants. More specifically, the paper examines the relation between grant size for Centres of Excellence (CoE), funded by the Danish National Research Foundation (DNRF), and various research performance measures, including productivity and impact. CoEs and their Principal Investigators funded by the DNRF are perceived to be among the absolute “elite” in the Danish (and international) science system. The DNRF was established in 1991 with the goal to fund the “best” research talents in the country; the first CoEs were funded in 1993. As of June 2015, 100 CoEs have been funded. Parent research institutions are expected to contribute and, after the expiration of the funding period, CoEs are expected to be nested within the host department(s), with the intention of securing CoE’s research environment in the longer term. Initially, centres are funded for a five-year period, whereafter the centres can apply for an additional five year extension; so that most centres end up with a 10-year funding period (recently this has changed to an initial six-year period with the possibility of an additional four years). Consequently, for the period examined in this paper, CoEs are typically the largest publicly funded grant form in Denmark, though the CoEs’ size can vary. While funding the potentially “best” research obviously is a key rationale behind CoEs, an additional argument is that large grants create a critical mass that enhances research performance through greater collaboration, they are better able to attract top international researchers, and they provide access to ample research time and funds [4].

We have unique performance data for Danish CoEs for the period 1993 to 2011. These include annual grant amounts, lists of publications for each CoE and field-normalized citation indicators based on these publications. In addition, more detailed data are available for the sub-period 2005–2011 that covers additional funding that CoEs have obtained from other sources and the number and composition of scientific staff. Together these data sets enable subtle examinations of various aspects of high-level funding and its relation to performance.

More specifically, we examine the following questions. What is the role of size for research performance? Can we identify cumulative effects of CoEs and how are these related to grant size? What are the dynamics of CoE performance over time, is there an optimal time length? We will analyse different dimensions of research performance, including productivity, citation impact and the production of top, highly cited papers. The analysis will draw on both descriptive and econometric methods. In order to analyze dynamics over time, we employ a dynamic panel data model in the econometric analysis. We should note here however, that while we examine the dynamics of these CoEs, we do not conduct a formal, quasi-experimental analysis that seeks to measure the impact of the center grants on participating researchers or compares centers’ performance with a comparison. Our main reason for not doing this is data limitations. We are unable to construct a suitable comparison group for these centers, given their size and characteristics. Furthermore, we are not able to identify the members of each CoE (aside from the PI), which would otherwise have allowed us to compare researcher behavior and performance before and under the CoE grants. However, this option may be feasible in the future due to more comprehensive data collection on CoE activities. We discuss this further in the conclusion of this paper.

The paper is organized as follows: the next sections discusses in more detail the relationship between funding size and research performance based on previous research findings reported in the literature. In the subsequent data and methods sections we present our data sets, the indicators and measures applied, and the model specifications used. Thereafter we present the results, first a descriptive analysis of trends in performance for CoEs, an examination of the staff composition of CoEs and growth in funding and personnel over time, and the results of the econometric analysis of the relationship between size and the dynamics of research performance. Finally, we summarize and discuss our results in the discussion section.

## Review: On the Relation between Funding Size and Research Performance

Empirical evidence on the impact of project size for grants is fairly limited, though a larger number of analyses have been conducted concerning the size of research units or groups where groups are typically estimated based on publication collaborations. A general result for research groups appears to be that there exists a critical mass threshold of around 5–8 members in a research group, but with no conclusive evidence of economies of scale. Productivity increases with size and at a certain point may decline [13] [14]. In the bibliometric analysis by Seglen and Aksnes [15] of Norwegian microbiological research for the period 1992–1996, the authors find no correlation between group size and scientific productivity. A more recent analysis of size and performance for UK research units does not find evidence of a relation between size and performance, either in terms productivity or citation impacts [16].

Fortin and Currie [1] examine the individual performance of researchers as a function of grant size from the Natural Sciences and Engineering Research Council of Canada (NSERC) context. They reason that large grants would be more effective only if scientific impact increases as an accelerating function of grant size. Their conclusion is that impact is positively, but only weakly, related to funding. Researchers who received additional funds from a second federal (Canadian) granting council, were not more productive than those who received only NSERC funding. Impact was generally a decelerating function of funding. Impact per dollar was therefore lower for large grant-holders. According to Fortin and Currie [1], this is inconsistent with the hypothesis that larger grants lead to larger discoveries and they suggest, contrary to Hicks and Katz [2], that funding strategies that target diversity, rather than “excellence”, are likely to prove to be more productive. It is important to notice, that the NSERC Discovery grants examined are grants for individual researchers and not CoEs.

Ida and Fukuzawa [17] generally find a positive impact of centers of excellence on scientific productivity. They examine productivity for the “Japanese 21^st^ Century Centers of Excellence”, comparing publication and citation counts of center participants with a control group across eight fields. In comparing productivity before and after the grant (difference in difference) for the two groups, they found significant increases in publication counts within four out of eight fields (life sciences, humanities, medical sciences and mechanical engineering) and positive and significant increases in citation counts within three out of eight fields (life sciences, information sciences and medical sciences). In contrast, a significant negative result was found for citation counts within mathematics and physics.

Rogers et al. [18] examine the impacts of NSF funded Nanoscale Size and Engineering Centers (NSEC). They find in general that centers play an important role in creating critical mass and networks through collaboration. They argue that NSECs have been crucial in coordinating and facilitating collaboration, both among academic researchers and with industry. In terms of publication and citation results, they find that centers perform much better than the field as a whole. For example, median numbers of citations are typically two to four times higher for NSEC papers compared to all papers within the field. However, the analysis was unable to isolate effects that were due to centers, or to assess the value for money in terms of publication performance (which is only one of many goals for the centers).

An important element that can influence the development of CoEs is the composition and diversity of centers and how members interact within centers. Youtie et al. [19] use bibliographic coupling methods to create measures of “centerness” and how networking patterns among center members evolves over time.

The present paper contributes to this literature by examining the dynamics of CoEs both in terms of research performance and accumulation of resources, and the role of center size in these dynamics. The focus and design of our analysis is to a large degree guided by the data that is available to us and its strengths and weaknesses. These strengths includes comprehensive lists of publications for each CoE, which we have drawn on to construct field-normalised, citation based indicators, and annual data on center funding from DNRF. We also have data on additional funding that CoEs have obtained over the course of the grant period, along with data on CoEs’ staff composition, though only for the period from 2005 onwards. A key limitation of our data is that we do not have lists of the members of each CoE and are thus unable to compare research performance for members before and during center participation. Our inability to identify center members also means that we are unable to analyse how CoEs may have influenced their collaboration and networking activities.

## Data

The analysis here utilizes both administrative data for CoEs from the DNRF and bibliometric data covering publications linked to the CoEs. The analysis includes all CoEs established in the period 1993–2007 and for which adequate data on publications was available in the Web of Science (WoS) database. CoEs established in 2009 or later were excluded from the analysis due to insufficient data (no centre grants were awarded in 2008). Clearly, we need a citation databse in order to examine citation performance. WoS is chosen because of its better data quality and longer historical coverage compared to alternatives. As a result, a small number of CoEs (12) within the humanities, social and computer sciences were excluded from the analysis due to inadequate coverage in WoS, leaving in all 57 CoEs for the analysis (another 9 CoEs were started in 2009/10 and another 12 in 2015. These are not included in the analysis). We should emphasize that similar challenges would be present with alternatives to WoS because a majority of publications from these centres (humanities and social sciences) are Danish language publications; and the computer science proceedings literature is generally poorly covered in the main citation databses. Of the 57 centers included, 34–35 grants were fully completed by 2011, while 21–22 were still in operation.

The primary administrative data used in the analysis includes yearly funding amounts and lists of publications linked to each CoE. For the period 2005–2011, detailed data is available on additional funding that CoEs have been awarded and on the composition of CoE scientific staff (senior faculty, international guest faculty, postdocs, PhD students and scientific assistants). This reflects in part changes in reporting requirements implemented in 2007, where CoEs now report any additional funding that CoE members have obtained and also more detailed information (such as name, academic position and affiliation) on the researchers and other staff that are funded by the CoEs.

The unique bibliometric data set used in the present analysis comes from a recent research evaluation of the DNRF in 2013, covering publications linked to the 57 CoE funded from 1993 to 2011. 2011 is the latest year possible in order to maintain comparable citation windows of approximately 3 years for all publications. The bibliometric analyses are carried out in the Web of Science (WoS) and the journal articles have been identified and matched from publication lists provided by the individual CoEs (in total approximately 11,200 unique publications were identified); for detailed descriptions of the data collection, see Schneider and Costas [20] for details.

### Types of indicators

We use a number of standard bibliometric indicators of output and impact. We use the acronyms given by the Centre for Science and Technology Studies (CWTS) at Leiden University to designate the indicators. The same indicators (and the same database) are used in the Leiden Ranking (www.leidenranking.com). Publication types include research articles, reviews and letters, where the two former publication types have a weight of 1 and the latter a weight of 0.25. All analyses have been done with full counting. Despite recent claims that fractional counting is generally preferable (see e.g., Aksnes, Schneider and Gunnarsson [21]; Waltman and van Eck [22]), we argue that the special characteristics of our unit of analysis (CoE) makes full counting more relevant as it reflects the CoEs’ actual research participation It would be somewhat arbitrary to apply fractional counting at the institutional level since several institutions can be involved in CoEs. As it is, full counts show the absolute number of publications in the WoS database linked to a CoE in the period examined.

Citation impact of publications is measured by two complimentary citation indicators: Mean Normalized Citation Score (MNCS) and the Proportion of Publications among the top 10% of the most highly cited in the database (PPtop10%). All citation indicators are item-normalized according to publication type, publication year, and field-specific citation rates. This means that citation rates for each publication are compared to average citation rates for the same type of publications, in the same year, and for the specific research field, before they are aggregated to provide totals. This enables the comparison of so-called relative citation indicators across research fields, publication types and publication years. MNCS is the average number of citations of publications, normalized for field and publication year. An MNCS value of two for instance means that the publications have been cited at twice the average of their field and publication year. PPtop10% is the the proportion of publications that, compared with other publications in the same field and in the same year, belong to the top 10% most frequently cited.

Such relative indicators are needed here because the typical number of citations is highly dependent on research field, publication type and the time allowed before citations are counted. Self-citations are excluded from the calculation of citation rates and citation rates are calculated with four-year citation windows, i.e., the citations obtained during the publication year and the following years are counted. For the most recent publications, citations have only been accumulated during two years.

An important weakness of the MNCS indicator is its strong sensitivity to publications with a very large numbers of citations. Especially for smaller numbers of publications this can result in an overestimation of the actual impact of the publications assigned to the unit of analysis. As the PPtop10% indicator is based on ranks and not averages, it is much less sensitive to publications with a very large number of citations. By default we apply 10% as the threshold for the indicator, where 10% means all publications cited on or above the 90^th^ percentile in the database. Obviously, such as a threshold creates an artificial dichotomy between publications that are respectively above and just below the percentile threshold. Therefore we apply both MNCS and PPtop10% as they can be seen as complementary, though they usually also correlate strongly at aggregated levels.

## Results

We first perform a statistical analysis of the trends in research performance over the course of grant periods for CoEs and across different size classes. Thereafter we conduct an econometric analysis of the dynamics between size, time and research performance.

**Fig 1:**
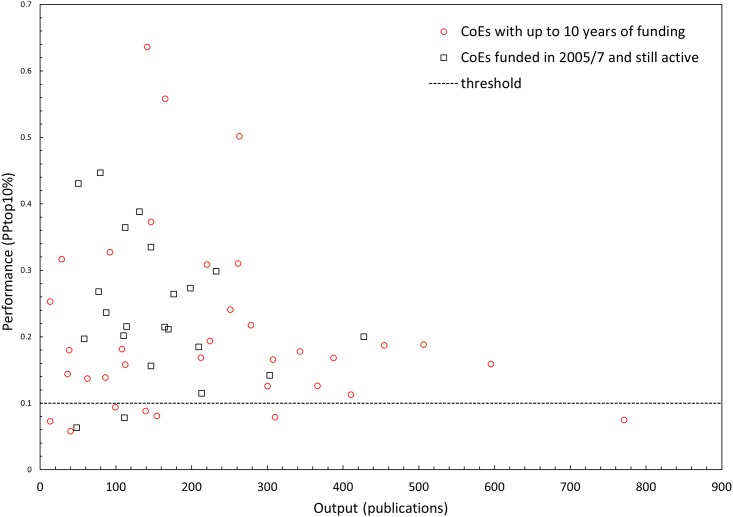
Performance variation of 57 CoE included in the bibliometric analyses of publications linked to the DNRF in the period 1993 to 2011. Circles indicate CoEs with full or terminated funding period at the time of the bibliometric analyses (i.e., 2013), and square indicate still active CoEs. Performance is measured with the PPtop10% indicator developed by CWTS, Leiden University, the Netherlands, and it measures the proportion of papers from a CoE that are among the 10% most cited in the Web of Science citation database; the expected proportion is 10% indicated with the dotted line.

The DNRF and its main funding instrument were evaluated by an international panel in 2013 (http://www.ft.dk/samling/20141/almdel/fiv/bilag/13/1413188.pdf). The empirical evidence comprises among other things bibliometric performance analyses. When considered as one unit, the performance of all publications linked to DNRF funded CoEs show performance levels with mean normalized citation scores (MNCS) around 2.07 and PPtop10% scores (the proportion of papers from a CoE that are among the 10% most cited in the Web of Science citation database) above 25% for the whole period. Fig 1 shows the variation in performances for 57 CoEs included in the bibliometric analyses. It is important to notice that the funding period for some of CoEs was expired at the time of the analysis, while funding for other CoEs were still running or even in its early phases. There is considerable variation in output and performance among the individual CoEs, some CoEs perform extremely well, while around 12–13% only perform at average international levels for the PPtop10%.

### Trends in performance for CoEs

Table 1 shows average trends in performance over time for the CoEs. Surprisingly, performance is highest in the first year of existence for CoEs. However, only 41 out of 57 CoEs have registered publications in the first year of their existence, which may have biased these first year results upwards. Overall mean MNCS for these 41 CoEs is 2.24 compared to 1.55 for the remaining CoEs. However, this is only part of the explanation as mean values for these 41 CoEs in the first year are still greater than averages overall (2.47 compared to 2.24). Research results that are already published in the first year of centre operations are also likely to have begun prior to the centre, and thus it is not clear how these first year results should be compared to results in the remaining years.

**Table 1:** Developments in annual measures of citation impact and productivity over time for CoEs.

		MNCS		Number publications		Number highly cited (top10%) publications		Number highly cited (top10%) publications per MN€		PPtop10%	
Grant year	Number CoEs	Mean	Median	Mean	Median	Mean	Median	Mean	Median	Mean	Median
1	41	2.47	1.65	12.66	10	3.902	3	3.16	2.42	31.0%	30.0%
2	57	1.94	1.61	16.35	15	4.436	3	3.79	3.09	28.4%	26.7%
3	57	2.00	1.51	20.39	20	5.053	4	4.45	3.67	25.9%	21.9%
4	57	1.72	1.47	22.62	20	5.436	4	4.60	3.89	23.9%	20.8%
5	56	1.88	1.44	25.04	23	6.107	5	4.90	3.98	24.4%	23.3%
6	47	2.64	1.43	27.11	24	6.426	5	5.42	4.64	26.8%	22.2%
7	40	1.93	1.51	29.15	22	6.7	5.5	5.57	4.78	24.9%	21.1%
8	27	2.11	1.70	29.00	29	6.963	6	6.33	5.11	27.1%	22.6%
9	27	2.21	1.57	34.37	31	8.259	6	7.34	6.15	24.8%	20.0%
10	24	1.97	1.43	30.08	26	7.458	6.5	6.91	4.77	28.1%	21.7%
Total	57	2.07	1.52	23.54	20	5.811	5	4.97	3.96	26.4%	22.7%

There does not appear to be any clear pattern over time for the citation measure, MNCS. The mean value of MNCS peaks in year 6 at 2.64; however the median value of MNCS is actually at its lowest in year 6 and peaks in year 8. Results over time are also similar for the share of top 10% publications. For all years, the mean value of MNCS is larger than the median, however the difference between the mean and median value varies from year to year and is largest in year 1 and year 6. This appears to be due in part to extreme values. If we consider MNCS values over 5, there are 5 values in year 6 (3 of which are over 10), none in year 4, one in year 10 and 2–4 values in the other years. While year 6 clearly has the greatest share of extreme values, and years 4 and 10 the lowest, there is no clear difference among the other years.

Differences in publication behavior (including average volume) across fields introduce bias to indicators based on the number of publications. However, to gain an overall idea of the trends in the volume of publications over time, we include both measures of the number of publications and number highly cited (top 10%) publications per million euros of CoE funding (ie. only DNRF centre funding). Developments in these numbers are also helpful in putting our two main field normalized citation based measures in perspective.

The number of publications per million euros of CoE funding shows strong signs of increasing over grant periods, by around 280% from year 1 to year 9, where publications per million euros peaks. Note again that these measures are only based on core CoE funding, and do not include any additional funding that the CoEs may have obtained over time. As will be shown below, DNRF CoE have been successful in obtaining a significant amount of funding from other sources, with this additional funding growing over the CoEs’ grant period. This would appear to be a key factor behind the large increases in number publications shown in Table 1. Finally, the production of top 10% articles also increases strongly over time, more than doubling from year 1 to year 9.

Note that the sample of CoEs for upon which these values are based is not the same over the entire period. Grant years 1 to 4 include all 57 CoEs, but data is available for only a subset for the remaining years. This is partly due to the fact that some CoEs are still in operation, while others only ran for a single five year period. However, if we were to hold the sample constant over time, for example including only the 24 CoEs with data for all ten years, then the results are qualitatively the same as those shown in Table 1; ie. there is no clear pattern over time for MNCS and PPtop10%, while both the number of highly cited publications and publications overall increase over time.

In order to examine eventual patterns in publication and citation activity according to grant size, individual annual grants are grouped in four quartiles according to size, based on average annual grant size. Both mean and median values for performance measures are shown in Table 2. Mean values for both MNCS and PPtop10% are highest for the third quartiles according to size. Average MNCS for these grants is 2.65 while PPtop10% is 32%. Median values for both performance measures are increasing in size, though the median for MNCS is highest in the third quartile, while the median for PPtop10% is highest for the fourth quartile. These statistics can thus give the impression that performance is increasing in grant size, though are also some signs of a possible non-linear relationship between size and performance, with citation impact increasing in size up to a certain point and thereafter falling beyond this threshold. We examine this more formally in the multivariate analysis below. Note also that, as for Table 1, mean values of MNCS can be influenced by extreme values, which appears to be the case for the first and third quartiles. For example, there are in all 26 values where MNCS is greater than five. Nine of these are in the first quartile, none in the second, 14 in the third, and 3 in the fourth quartile.

**Table 2:** Overall measures of citation impact and productivity for CoEs by annual grant size (in MN€).

	MNCS		PPtop10%	
Grant size	Mean	Median	Mean	Median
Q1 (<0.864 MN €)	2.024	1.281	0.23	0.20
Q2 (0.864–1.102 MN€)	1.595	1.392	0.21	0.20
Q3 (1.102–1.456 MN€)	2.654	1.758	0.32	0.25
Q4 (> 1.456 MN€)	1.957	1.724	0.30	0.27
Total	2.069	1.517	0.26	0.23

**Fig 2:**
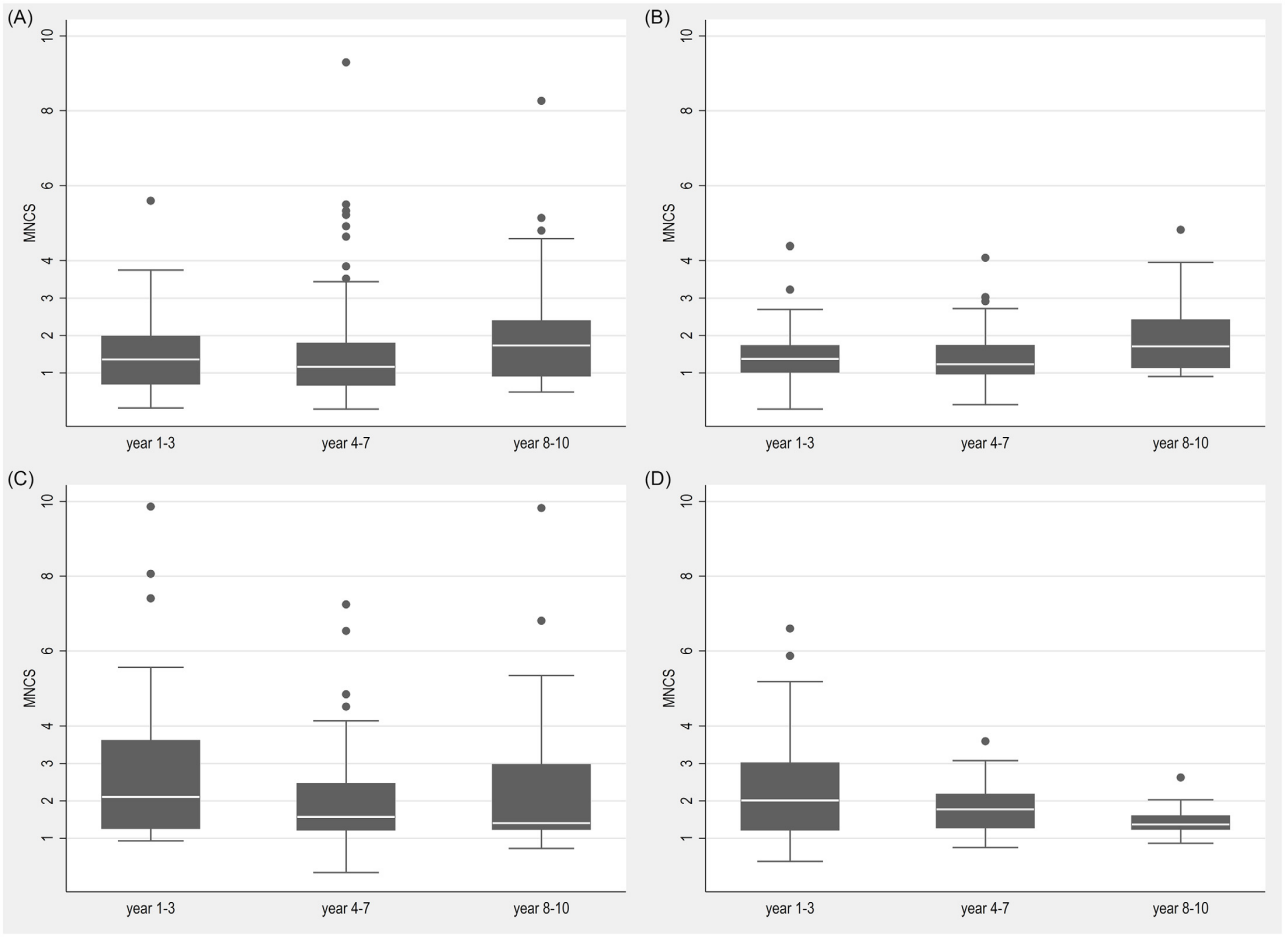
Distributions of MNCS across grant years and size classes. Fig 2.a. Q1 (<0.864 MN €). Fig 2.b. Q2 (0.864–1.102 MN€). Fig 2.c. Q3 (1.102–1.456 MN€). Fig 2.d. Q4 (> 1.456 MN€).

However, the statistics in Tables 1 and 2 are not fully able to show how performance indicators are distributed across both time and size. Fig 2 below attempts to examine both of these dimensions. In Fig 2, we display box plots of MNCS scores over the course of grant periods for these four size classes. Given the relatively small number of observations per year, ranging from 24 to 57, we have grouped grant years into three periods: years 1 to 3, years 4 to 7, and years 8 to 10. For illustrative purposes, all values over 10 have been removed from these figures. The picture from Fig 2 is somewhat different from that for Tables 1 and 2. In general, it appears that MNCS is falling over the course of grant periods for the largest CoE grants, while it is increasing for those among the smallest half. There does not seem to be any pattern over time for CoE grants in the third quartile, which though have had fairly high performance throughout.

Statistical tests generally support these indications. Note that while values over 10 are not shown in Fig 2, these extreme values are included when performing the statistical tests. We have conducted groupwise (Kruskal-Wallis, KW) and pairwise (Mann-Whitney, MW) non-parametric tests comparing the distributions of observations for the three periods. For the smallest quartile and the third quartile, no significant differences were found between the three periods (KW-p-value for Q1 is 0.362 and for Q3 is 0.519). For the second quartile, however, values in the third period were found to be significantly higher than both the first and second periods (p-values of 0.08 and 0.02 respectively). For the largest quartile, no significant difference was found between the first and second periods (MW-p-value = 0.31), but both these periods were found to be higher than the third period (MW-p-values of 0.09 and 0.07). The p-value for the groupwise Kruskal-Wallis test for Q4 is 0.123.

While smaller CoEs tended to increase more in performance over time, it is still the case that overall performance was higher for larger CoEs, as Table 2 above indicates. Over the first two periods, values in the largest two quartiles are significantly higher than those in the smallest quartiles (in all cases, p-values were less than 0.02), while there is no significant difference among size classes in the third period (groupwise KW-p-value is 0.699). While not shown here, the pattern is quite similar for PPtop10%.

### Funding accummulation over time

In this section, we examine more closely external funding and the research staff composition of CoEs, both of which can help illuminate patterns of accumulation in CoEs. Given that this data is only available from 2005 onwards, we only have data for a subset of CoEs (20) and generally only for part of these CoEs’ grant periods. For this reason, these variables are not included in the econometric analysis below, and are used here only, in order to provide a descriptive picture over time.

Table 3 shows key variables over time for this subset of CoEs that have started between 2005 and 2007. On average, 37 scientific personnel were employed by the CoEs in the seven years covered by this data, 26 in terms of full-time equivalents. Scientific personnel consisted of 8 senior faculty, 2 visiting international faculty, 10 postdocs, 15 PhD students and 2 scientific assistents (Administrative personnel were not included in these calculations.). As is shown in Table 3, the average number of scientific staff increases substantially over these 6 years, from 27 to 44, amounting to an increase of 61%. The bulk of this increase is in PhD students and postdocs. In terms of percentage increases, the largest increases are for international guest faculty and scientific assistants, though these numbers are still fairly low in absolute terms.

**Table 3:** Developments in scientific staff and additional funding over time for CoEs.

Grant year	Scientific staff	Scientific FTEs	Faculty staff	Visiting staff	Postdoc staff	PhD staff	Assistent staff
**1**	25.4 (8–43)	13.2	7.4	0.7	7.6	9.5	0.4
**2**	33.5 (14–61)	24.7	7.6	1.3	9.9	13.3	1.4
**3**	37.4 (14–77)	27.8	7.8	2.2	10.9	14.9	1.7
**4**	41.1 (15–83)	30.1	8.1	1.8	11.3	17.6	2.4
**5**	43.2 (17–73)	30.7	8.6	2.5	12.6	17.1	2.4
**6**	42.2 (20–69)	28.8	8.0	2.5	11.5	17.2	3.0
**7**	40.5 (14–69)	29.8	8.1	1.8	11.3	16.7	2.6
Total	37.2	26.1	7.9	1.8	10.6	15.0	1.9
Grant year	Additional funding (MN€)	Additional funding (in % term grant)	Additional funding (in % term grant) large CoEs	Additional funding (in % term grant) Small CoEs			
**1**	0.63	11%	11%	11%			
**2**	0.81	13%	15%	9%			
**3**	0.93	16%	16%	16%			
**4**	1.18	20%	19%	26%			
**5**	1.10	19%	19%	22%			
**6**	1.46	28%	29%	24%			
**7**	1.30	25%	26%	22%			
Total	1.04	18%	18%	19%			

Data on scientific staff and additional funding only available from 2005 onwards. Mean values shown for all variables. For total scientific staff, max-min range shown in parentheses.

CoEs have thus expanded over time, while yearly DNRF grant amounts have been relatively constant over grant periods. This expansion has instead been financed by additional grants secured by the CoEs. Table 3 shows the amount of additional funding secured each year as a share of 5 year term CoE grants. Over the period, CoEs secured on a yearly basis additional funding that amounted 18% of CoE term (5 year) grants on average. In other words, over the course of a five year grant term, CoEs were typically able to accumulate additional funding that almost matches actual funding from the CoE grant itself. In total, 55% of additional funding stemmed from other public sources in Denmark, 26% from Danish private funds, and 19% from international sources such as the EU Framework Programmes and the ERC. Hence, CoEs have been able to accumulate a substantial amount of additional funding over grant periods, which primarily has gone towards junior researchers. There thus appears to be a generational dimension to these accumulative effects; funds are concentrated in these elite units, but are at the same time allocated more widely to early career researchers. While we are unable to examine this aspect further here, it would be very interesting to follow how center participation influences the performance and career paths of these early career researchers. We reflect on this point in more detail in the conclusion of the paper.

We have also examined whether grant size plays any role in this accumulation. Table 3 shows additional funding for CoEs that are above and below the median grant size. There is no clear difference in annual shares between larger and smaller CoEs, with smaller CoEs highest in the fourth and fifth years while larger CoEs have higher shares in the sixth and seventh grant years. However, it should be kept in mind that these figures are based on a fairly small number of CoEs and that they only cover part of their overall grant periods.

## Dynamic Panel Data Analysis

As is clear from the discussion above, the relation between funding size and research performance is complex and may potentially be influenced by a number of factors. Size may have a positive impact on performance if it facilitates research or research collaborations that would not have been fully possible otherwise, or at least not to the same degree. There may also be a dynamic element to new research groups or units created through grants. In particular for large centres, it may take time for researchers to capitalize on synergies with other researchers, both through informal interaction and through active collaboration on papers or specific projects. This would argue that research performance is increasing over time for CoEs. However, at the same time, there is a possibility that these potential benefits may decrease over time, which would imply that there may be some point at which CoEs ‘peak’ in terms of performance. Both size and time arguments suggest that there is an accumulative element here, where critical mass helps create conditions for good research and where researchers are able to build on previous research performance.

Our aim here is to employ multivariate analysis to estimate the impact of size and time on research performance. Our choice of approach, dynamic panel data models, is driven by a number of factors related to the data, the objective of our analysis and potential relations between variables. First, it seems very likely that performance is dependent on past levels of performance; i.e. any effects related to grant size or duration are likely conditional upon the ability and past performance of CoEs. Some center-specific factors could essentially be considered to be constant over the course of the grant period while others can be expected to evolve over time.

Second, research production is a dynamic process, where the time from initiation of research to actual publication can vary greatly from project to project. These lagged performance variables can though also potentially be affected by grants in the same period and the performance variables can also influence each other. This also implies that these variables will likely be correlated with the error terms in equations, which introduces bias to coefficient estimates. The solution here is to find variables (‘instruments’) that are highly correlated with the explanatory variables but uncorrelated with error terms.

Third, we have longitudinal data (or panel data) with a fairly large number of ‘individuals’ (in our case, CoEs) and a fairly short time series, with 10 years or less of data for each CoE. In order to take account of both the endogeneity of these explanatory variables and the panel structure of the data, where we follow a cross-section of CoEs over time (and where the lagged dependent variable is included in regressions as an explanatory variable) the model is estimated using a dynamic panel data model, that was designed for use concerning data and problems that are similar to those here [23] [24]. We model the impact of size (in terms of grant size and number of publications) and time on research performance in the following way. First, we examine two different measures of research performance: 1) MNCS and 2) the share highly cited articles (top 10%). Size is measured by the annual DNRF grant amount (in million euros) and by the annual number of articles. The number of articles provides a further measure of critical mass. On perspective on these two measures is that the grant amount measures the potential for creating a critical mass while the number of publications provides an indication of the extent to which it actually comes about. To allow for the possibility of decreasing returns to size, the square of annual grant amounts is also included. Accumulation of knowledge and expertise through earlier performance is captured through the lag of productivity of highly cited articles and the lag of the dependent variable in question. Finally, a variable for the grant year and its square are included to capture trends in performance over the grant period, and also allow for possible decreasing returns over time. For pragmatic purposes, we choose a simple straightforward structure where all explanatory variables are lagged one period (so, performance is measured in time t and all explanatory variables in time t-1).

The model we estimate is thus the following:

$$y_{i,t}=\rho y_{i,t-1}+{x^{\prime }}_{i,t-1}\beta +{z^{\prime }}_{i,t-1}\gamma +\alpha_{i}+\varepsilon_{i,t}$$

where

i = 1..N is an index for CoEs

t = 1..T is an index for time

y_i,t_ is the dependent variable, either 1) MNCS and 2) the share highly cited articles (top 10%)

x_i,t-1_ is a row of explanatory variables concerning the CoEs research activity in period t-1 (number articles, number highly cited articles per MN€ of CoE funding, share articles with international co-author)

z_i,t-1_ is a row of explanatory variables concerning CoE grant funding in period t-1 (grant year, grant year squared, grant amount, grant amount squared).

We assume that the lagged performance variables (MNCS, PPtop10%, number publications and productivity of highly cited articles) are endogenous and we thus need instruments to obtain unbiased estimates of the effects of these variables. We estimate a systems Generalised Method of Moments (GMM) model [25], using lags both in levels and in differences as instruments for the explanatory performance variables in the model. The validity of this approach is thus dependent on the relevance of the instruments used. Two tests are commonly used to shed light on this issue. The Hansen test [26] essentially examines whether variables used are valid instruments (and thus are not correlated with the error term). The test is though weakened by a large number of instruments, though is not affected by heteroskedasticity. The related Sargan test [27] is not affected by the number of instruments but is affected by heteroskedasticity. Hence, there is a trade-off or complementarity between the two tests of specifications. If the tests are not rejected (ie. p-values not below a critical threshold) then this gives us an indication that the variables used work adequately as instruments for the (endogenous) explanatory variables in the model. We can see from Table 4 below that this is the case for both regressions.

**Table 4:** Results of the dynamic panel data regressions.

	(1)		(2)	
VARIABLES	MNCS		PPtop10%	
	Coeff.	P-value	Coeff.	P-value
	(std. err.)	(95% CI)	(std. err.)	(95% CI)
MNCS (t-1)	0.326***	0.001		
	(0.088)	(0.149–0.502)		
Number articles (t-1)	-0.026***	0.010	-0.000	0.730
	(0.010)	(-0.045 - -0.006)	(0.001)	(-0.002–0.002)
No. highly cited articles	0.107***	0.004	0.002	0.706
per MN € (t- 1)	(0.036)	(0.035–0.178)	(0.004)	(-0.007–0.011)
Grant_year (t-1)	0.296*	0.094	0.008	0.615
	(0.174)	(-0.052–0.644)	(0.016)	(-0.024–0.040)
Grant_year_sq (t-1)	-0.021	0.170	-0.000	0.724
	(0.015)	(-0.053–0.010)	(0.001)	(-0.003–0.002)
Grant amount (t-1)	2.966***	0.007	0.071	0.431
	(1.068)	(0.828–5.105)	(0.089)	(-0.108–0.249)
Grant amount sq (t-1)	-1.001**	0.010	-0.022	0.421
	(0.378)	(-1.758 - -0.244)	(0.028)	(-0.078–0.033)
Share articles with intl.	-1.174	0.141	-0.012	0.851
co-authors (t-1)	(0.787)	(-2.749–0.402)	(0.065)	(-0.143–0.119)
Share PPtop10% (t-1)			0.648***	0.000
			(0.132)	(0.382–0.913)
Constant	-0.739	0.170	0.019	0.709
	(0.532)	(-1.805–0.327)	(0.052)	(-0.085–0.124)
Observations	370		370	
Number CoEs	57		57	
Sargan test of overid. restrictions (p-value)	0.821		0.958	
Hansen test of overid. restrictions (p-value)	1.000		1.000	

Estimation method: systems GMM. Lags (t-2 and t-3) of MNCS, PPtop10%, number articls no. highly cited articles per MN€ and grant amount used as instruments (both in levels and differences).

*** p<0.01

** p<0.05

* p<0.1

The first column of Table 4 shows results for the MNCS. The estimated coefficient of grant years is 0.296 (p-value = 0.094) while the coefficient for its square is -0.021 (p-value = 0.17). This could thus potentially be seen as weak evidence of a time element for grant performance. 95% confidence intervals for grants years range from -0.052 to 0.644 and from -0.053 to 0.01 for its square. These coefficients imply that, controlling for other factors, performance is on average highest at a grant length of 6.7 years (I.e. by taking the derivative of 0.296*grant years -0.021*grant years^2^ and setting equal to zero, we find the number of grant years that maximizes impact on MNCS.).

Concerning grant size, grant amounts are positive while its square is negative. P-values of coefficient estimates for both variables are 0.01 or lower, implying that 95% confidence intervals for grant amounts (in MN euros) are fully positive while those for its square are fully negative. This thus indicates that citation impact is increasing with grant size, but at a decreasing rate. As with time, we can calculate the annual grant size for which performance is highest based on these coefficient estimates, which gives 1.45 MN euros.

Previous citation impact (t-1) is positively correlated with current MNCS, with a positive coefficient of 0.326 (p-value = 0.001). In addition to past MNCS (t-1), productivity of highly cited articles is also positive with a p-value = 0.004. Hence, past performance (t-1) both in terms of average citation impact and in terms of productivity of highly cited articles are important determinants of current citation impact. On the other hand, the relation to the number of articles overall is negative (p-value = 0.01).

Results are quite different for the share of highly cited articles, PPtop10%. In contrast to MNCS, only the lagged dependent variable appears to have an effect for PPtop10%, with a positive coefficient of 0.648 (p-value = 0.000). None of the other variables can be considered significant at any threshold, with p-values 0.40 or higher in all other cases. Drawing also on the results above in Table 4, it would appear that large grants generate a larger number of highly cited articles, but do not have a significant effect on their share relative to the number articles overall.

## Discussion

This paper has investigated the relation between size, accumulation and performance for research grants, where we have examined the relation between grant sizes for Centres of Excellence (CoE) funded by the Danish National Research Foundation (DNRF) and various ex post research performance measures, including impact and shares of highly cited articles. In doing so, we have examined both the relationship between size and performance and also how performance for CoEs evolves over the course of grant periods. This final section summarizes and discusses the results of the analysis.

In general, performance for these CoEs has been very high. The average MNCS for the 57 CoEs covered in the analysis is 2.07, which implies a field normalized citation impact that is more than double world (or WoS database) averages. The share of highly cited (top 10%) articles is also very high, with an overall share of 26% of articles among the top 10% most cited within their respective field. However, it should be noted once again that this performance does not necessarily reflect the effects of these CoEs. In particular, we are unable to distinguish between effects of the individual researchers (ie. that would have been generated in the absence of the CoE grant) and effects of the centers themselves.

The CoEs have been very successful in securing additional funding, which can be viewed as a ‘cumulative effect’ of center grants. Over the course of grant periods, the CoEs have obtained additional funding amounts that nearly match the size of centre grants themselves, which in turn leads to increases in the number of researchers. In terms of new personnel, the far majority of additional funding is spent on early career researchers, both postdocs and PhD students. Hence, this accumulation would appear to have a ‘generational’ dimension, allowing for scientific expertise to be passed on to an increasing number of younger researchers. The fact that levels of research performance are maintained over the course of this accumulation suggests that this generational transfer is successful.

When examining overall performance over the course of grant periods, larger CoEs have higher performance both measured in citation impact (MNCS) and shares of highly cited articles (PPtop10%), though there appears to be some indication that performance peaks and begins to fall for the largest CoEs. However, the picture is somewhat different when we examine performance over time. In general, it appears that both MNCS and PPtop10% are falling over the course of grant periods for the largest CoE grants, while it is increasing for those among the smallest half. Hence, while performance is quite high for the largest CoE grants, particularly in initial years, performance appears to increase most for smaller CoEs. In fact, for the final year of grant periods, the median value for MNCS is highest for the smallest grants. A possible explanation for this pattern is that there may be greater coordination costs connected with the largest CoEs, which slows their growth in performance.

At the same time, these general descriptive statistics may be unable to capture the dynamics in accumulation and research performance for individual CoEs. In order to examine these relations in more detail, we conducted a multivariate regression analysis of the role of size and time for research performance, also taking into account past performance of CoEs. These dynamic panel data analyses find evidence that performance, as measured by average citation impact, is increasing in grant size and over time. In both cases, the relation appears to be non-linear, suggesting that there is a point at which performance peaks. Concerning grant size, grant amounts are positive while its square is negative. P-values of coefficient estimates for both variables are 0.01 or lower, implying that 95% confidence intervals for grant amounts (in MN euros) are fully positive while those for its square are fully negative. This thus indicates that citation impact is increasing in grant size, but at a decreasing rate. We use these coefficients to calculate an estimate of the optimal annual grant size, which gives 1.45 MN euros. Though, it should be noted that these are general trends that do not take into account any potential differences across fields. For example, the need for expensive equipment or materials would clearly influence these size considerations. Results are weaker, but suggest a similar non-linear relation with respect to time. A similar estimate for grant years suggests that research performance in terms of MNCS peaks at 6.7 years.

These findings contradict those of Fortin and Currie [1], although it is very important to recognize that they examined relatively smaller NSERC grants to individuals, whereas we examine large-scale CoEs with long-term generous funding. As Fortin and Currie [1] point out themselves, the answer to the “question does greater funding of high performers lead to greater scientific impact, versus funding more researchers” depends upon the goals of the funding program. In the case of Danish CoEs the goal to “maximize discoveries” by focusing on “excellence” seems to work. And one thing to notice, numerous younger researchers are involved with a CoE over time, in that process some perhaps many of them will experience “cumulative effects” when it comes to recognition and this will serve them well in the future.

Over the course of developments in CoEs over time, with significant growth in researcher staff, PPtop10% remains fairly constant. Results from the regressions show a high degree of autocorrelation with a coefficient of 0.65 for the lagged value of PPtop10% and no effect of grant size or length. One potential interpretation of this is that CoEs or perhaps the smaller research groups within CoEs are able to transfer or maintain their research capabilities to new and typically less experienced researchers, with the result that production of highly cited articles increases parallel to increases in publication overall.

In the introduction we discussed the ‘cumulative effects’ within science and the potential adverse effects of this accumulation. While we are unable to discern from our analysis what is driving accumulation, eg. whether it is reputation of individual researchers or of the CoE or if it is grounded in the research results themselves, it seems clear from the data that these CoE grants lead to a further accumulation and concentration of resources. It thus also seems reasonable to assume that this accumulation contributes to existing inequalities among senior researchers, though the scope of this depends to a great degree on how large centre grant funding is relative to other forms of funding. At the same time, it is important to again note that this accumulation of funding often goes towards an increase in the funding of younger researchers.

We have noted above in this paper some limitations of this analysis, in particular concerning our ability to formally analyze the effects of CoEs on performance and also on collaboration and network patterns in general. These would constitute important topics for future research and should be feasible given the DNRFs new reporting requirements implemented in 2007. Hence, in a few years time, it will be possible to analyze a cohort of CoEs over their full grant period with the new, expanded data. Provided that researchers can be identified, research performance could be measured both before and during CoE participation. Generational effects would also be of great interest here, in order to analyze to what extent early career researchers benefit from their participation in CoEs and working under top researchers within their field. An additional topic of interest is the composition of CoEs in terms of established vs. junior researchers, national vs. international researchers, research field diversity, and how this influences research performance and collaboration.

## References

[1] Fortin J. M., & Currie D. J. Big Science vs. Little Science: How Scientific Impact Scales with Funding. *PLoS ONE*, 8(6); 2013; doi: 10.1371/journal.pone.0065263 10.1371/journal.pone.0065263 PMC3686789 23840323

[2] Hicks D., & Katz J. Equity and Excellence in Research Funding. *Minerva*: 49(2); 2011; p. 137–151.

[3] Jamtveit B., Jettestuen E., & Mathiesen J. Scaling properties of European research units. *Proceedings of the National Academy of Sciences*. 106(32); 2009; p. 13160–13163. 10.1073/pnas.0903190106 PMC2714277 19625626

[4] Bloch C. & Sørensen M.P. The size of research funding–trends and implications. *Science and Public Policy* 42(1); 2015; p. 30–43.

[5] Walsh J.P. & Lee Y-N. The bureaucratization of science. *Research Policy*; 44(8); 2015; p. 1584–1600.

[6] Merton R.K. The Matthew Effect in science. *Science*: 159(3810); 1968; p. 56–63. 5634379

[7] Cole J. R., & Cole S. Ortega Hypothesis. *Science*: 178(4059); 1972; p. 368–375. 17815351 10.1126/science.178.4059.368

[8] de Solla Price D. J. A general theory of bibliometric and other cumulative advantage processes. *Journal of the American Society for Information Sciences*. 27(5); 1976; p. 292–306.

[9] Allison P. D., Long J. S., & Krauze T. K. Cumulative Advantage and Inequality in Science. *American Sociological Review*; 47(5); 1982; p. 615–625.

[10] Stephan P. The Economics of Science. *Journal of Economic Literature*; 34; 1996; p. 1199–1235.

[11] DiPrete T. A., & Eirich G. M. Cumulative Advantage as a Mechanism for Inequality: A Review of Theoretical and Empirical Developments. *Annual Review of Sociology*. 32; 2007; p. 271–297.

[12] Hermanowicz J. The Culture of Mediocrity. *Minerva*. 51(3); 2013; p. 363–387

[13] von Tunzelmann N., Ranga M., Martin B. and Geuna A. *The Effects of Size on Research Performance*: *A SPRU Review*. Report prepared for the Office of Science and Technology, Department of Trade and Industry: 2003.

[14] Johnston R. Effects of resource concentration on research performance, *Higher Education* 28; 1994; p. 25–37.

[15] Seglen P.O. and Aksnes D.W. Scientific productivity and group size: A bibliometric analysis of Norwegian microbiological research, *Scientometrics* 49(1); 2000; p. 125–143.

[16] University Alliance. *Funding research excellence*: *research group size*, *critical mass and performance* Report prepared by Evidence. London: University Alliance: 2011.

[17] Ida T. and Fukuzawa N. Effects of large-scale research funding programs: a Japanese case study, *Scientometrics* 94; 2013; p. 1253–1273.

[18] Rogers J., Youtie J. and Luciano K. Program-level assessment of research centers: Contribution of Nanoscale Science and Engineering Centers to US Nanotechnology National Initiative goals, *Research Evaluation*, 21; 2012; p. 368–380.

[19] Youtie J., Kay L. & Melkers J. Bibliographic coupling and network analysis to assess knowledge coalescence in a research center environment. *Research Evaluation* 22; 2013; p. 145–56.

[20] Schneider J.W. & Costas R. Bibliometric analyses of publications from Centres of Excellence funded by the Danish National Research Foundation Report to the Danish Ministry of Science, Innovation and Higher Education: 2013 http://ufm.dk/en/publications/2013/files-2013/appendiks-5_bibliometrisk_report_03122013.pdf.

[21] Aksnes D. W., Schneider J. W., & Gunnarsson M. Ranking national research systems by citation indicators. A comparative analysis using whole and fractionalised counting methods. *Journal of Informetrics*, 6(1); 2012; p. 36–43.

[22] Waltman L., & van Eck N. J. (2015). Field-normalized citation impact indicators and the choice of an appropriate counting method. *Journal of Informetrics*, 9(4), 872–894.

[23] Arellano Manuel and Bond Stephen. Some Tests of Specification for Panel Data: Monte Carlo Evidence and an Application to Employment Equations, *Review of Economic Studies*, 58; 1991; p. 277–297.

[24] Arellano M. and Bover O.. Another Look at the Instrumental-Variable Estimation of Error-Components Models, *Journal of Econometrics*, 68; 1995; p. 29–51.

[25] Roodman D. How to do xtabond2: An introduction to difference and system GMM. *Stata Journal* 9(1); 2009; p. 86–136.

[26] Hansen L. Large sample properties of generalized method of moments estimators. *Econometrica* 50(3); 1982; p. 1029–1054.

[27] Sargan J. The estimation of economic relationships using instrumental variables. *Econometrica* 26(3); 1958; p. 393–415.

